# Burden of respiratory syncytial virus (RSV) hospitalizations among adults aged 60 years or more in Brazil: A retrospective surveillance database analysis (2013–2024)

**DOI:** 10.1371/journal.pone.0357303

**Published:** 2026-09-18

**Authors:** Bruna Medeiros Gonçalves de Veras, Lessandra Michelin, Julio Croda, Thaís Zamboni Berra, Gustavo Pires-Matheus, Igor Sampietri, Magda Araujo, Rosemeri Maurici da Silva, Adriana Guzmán-Holst

**Affiliations:** 1 Fiocruz, Rio de Janeiro, Brazil; 2 GSK, Rio de Janeiro, Brazil; 3 School of Medicine, Federal University of Mato Grosso do Sul, Brazil; 4 Oswaldo Cruz Foundation, Mato Grosso do Sul, Brazil; 5 IQVIA, São Paulo, Brazil; 6 Universidade Federal de Santa Catarina (UFSC), Florianópolis, Brazil; 7 GSK, London, United Kingdom; Chung Shan Medical University, TAIWAN

## Abstract

**Background:**

Globally, respiratory syncytial virus (RSV) is a leading cause of severe respiratory illness in adults aged ≥60 years. Risk increases with advancing age and comorbidities, particularly cardiopulmonary disease and diabetes. RSV disease burden in adults is often underestimated due to non-specific presentation and limited testing. In Brazil, RSV is a recognized driver of hospitalizations and deaths in older adults, yet comprehensive epidemiological data remain scarce. Here, we describe RSV-associated severe acute respiratory infection (SARI) hospitalizations among adults aged ≥60 years in Brazil between 2013 and 2024.

**Methods:**

We retrospectively analyzed RSV-associated SARI hospitalizations reported to the national Influenza Epidemiological Surveillance Information System -Gripe. Outcomes included hospitalization frequency, in-hospital mortality, case fatality rates (CFRs), seasonality, healthcare resource utilization, and comorbidities.

**Results:**

Between 2013 and 2024, 4,636 RSV-related hospitalizations occurred in adults aged ≥60 years, increasing sharply after the COVID-19 pandemic (2020–2024). Hospitalization rates increased from <1 per 100,000 in 2013 to >15 per 100,000 in 2024. Women accounted for most cases (59%), and hospitalizations were concentrated in the South and Southeast regions partly due to test availability. In-hospital mortality increased over time, with mean rates nearly fivefold higher post-pandemic than pre-pandemic. CFRs remained consistently high (21–31%), particularly in adults aged ≥80 years. The mean length of hospital stay was 12.9 days. Almost one-third of cases required intensive care unit admission, of whom more than two-thirds needed ventilatory support. Comorbidities were present in 71% of cases, most commonly cardiovascular (65%) and diabetes (33%)were strongly associated with increased mortality and healthcare resource utilization.

**Conclusions:**

RSV poses a substantial and increasing burden among Brazilian adults aged ≥60 years, with a high CFR, prolonged hospitalizations, and intensive healthcare resource utilization. These findings highlight the need to strengthen RSV surveillance and discuss vaccination strategies for adults aged older in Brazil.

## Introduction

Respiratory syncytial virus (RSV) is a highly transmissible pathogen that causes a spectrum of diseases, ranging from mild infections to severe illness. Infants, adults aged ≥60 years (particularly those with comorbidities), and immunocompromised individuals remain particularly vulnerable to severe outcomes with RSV infection [1–4]. Globally, both in high and lower-income countries, RSV is a leading cause of severe respiratory illness in adults aged ≥60 years [5,6]. Disease severity and hospitalization rates increase with age [4], and adults aged ≥75 years account for a large proportion of these cases [7]. Severe outcomes, including admission to an intensive care unit (ICU), the need for mechanical ventilation, and mortality, occur notably more frequently among adults aged ≥65 years, particularly those with underlying medical conditions such as chronic obstructive pulmonary disease (COPD), congestive heart failure, diabetes, and obesity [8]. This context highlights the importance of preventive measures, including vaccination, for adults aged ≥60 years, who are at high-risk of severe RSV disease.

A further issue is that the RSV burden in adults is often underestimated due to non-specific symptoms, low viral loads, delayed diagnosis, and limited access or lack of testing [9]. Laboratory confirmation is ideally using RT-PCR or multiplex PCR panels, which are currently the most sensitive and widely used methods for diagnosing RSV across age groups; serological tests are not used for acute clinical diagnosis due to delayed antibody responses, and antigen-based methods have lower sensitivity, especially in adults. [10].

In Latin America, epidemiological data on RSV remain limited. Nevertheless, RSV is known to be a major driver of hospitalizations among children aged <5 years and adults aged ≥60 years, particularly those with comorbidities [9]. In Brazil, among children aged <5 years, RSV is the leading cause of hospitalizations for respiratory diseases. It accounts for 60–75% of bronchiolitis cases [11]. Given the lack of data due to underdiagnosis in adults aged >20 years, a modeling exercise was conducted; this estimated that RSV infection was responsible for 44,323 hospitalizations and 9,115 deaths in 2019 in Brazil, with between 60–74% of cases and 79–88% of deaths occurring in adults population [12]. In this group, case fatality rates (CFRs) exceed 30% [13].

In 2009, Brazil introduced the national Influenza Epidemiological Surveillance Information System (SIVEP-Gripe),which serves as a cornerstone of the national surveillance system for severe acute respiratory infections (SARIs) [14,15]. Managed by the Ministry of Health, it systematically captures data on hospitalized SARI cases across all federative units, including those attributed to respiratory viruses such as influenza, SARS-CoV-2, and RSV. The system includes demographic, clinical, laboratory, and outcome information, and has been progressively enhanced over time to improve completeness and diagnostic specificity. National surveillance data in Brazil also indicate increasing RSV-related SARI among older adults [16].

RSV epidemics follow seasonal patterns that vary by region. In temperate climates, peaks occur in winter. In Brazil, as a tropical country with prolonged circulation of RSV regardless of seasonality, a temporal peak is observed beginning in autumn and extending through winter, with variations across different regions: February-June in the North; March-July in the Northeast, Central-West, and Southeast; and April-August in the South [17–21]. During the COVID-19 pandemic and in the years since, these patterns have been disrupted. Non-pharmaceutical interventions deployed during 2020 and 2021 to control the spread of COVID-19 also suppressed the circulation of RSV. Subsequently, this contributed to a “triple epidemic” comprising RSV, COVID-19, and influenza [19,22].

**Fig 1:**
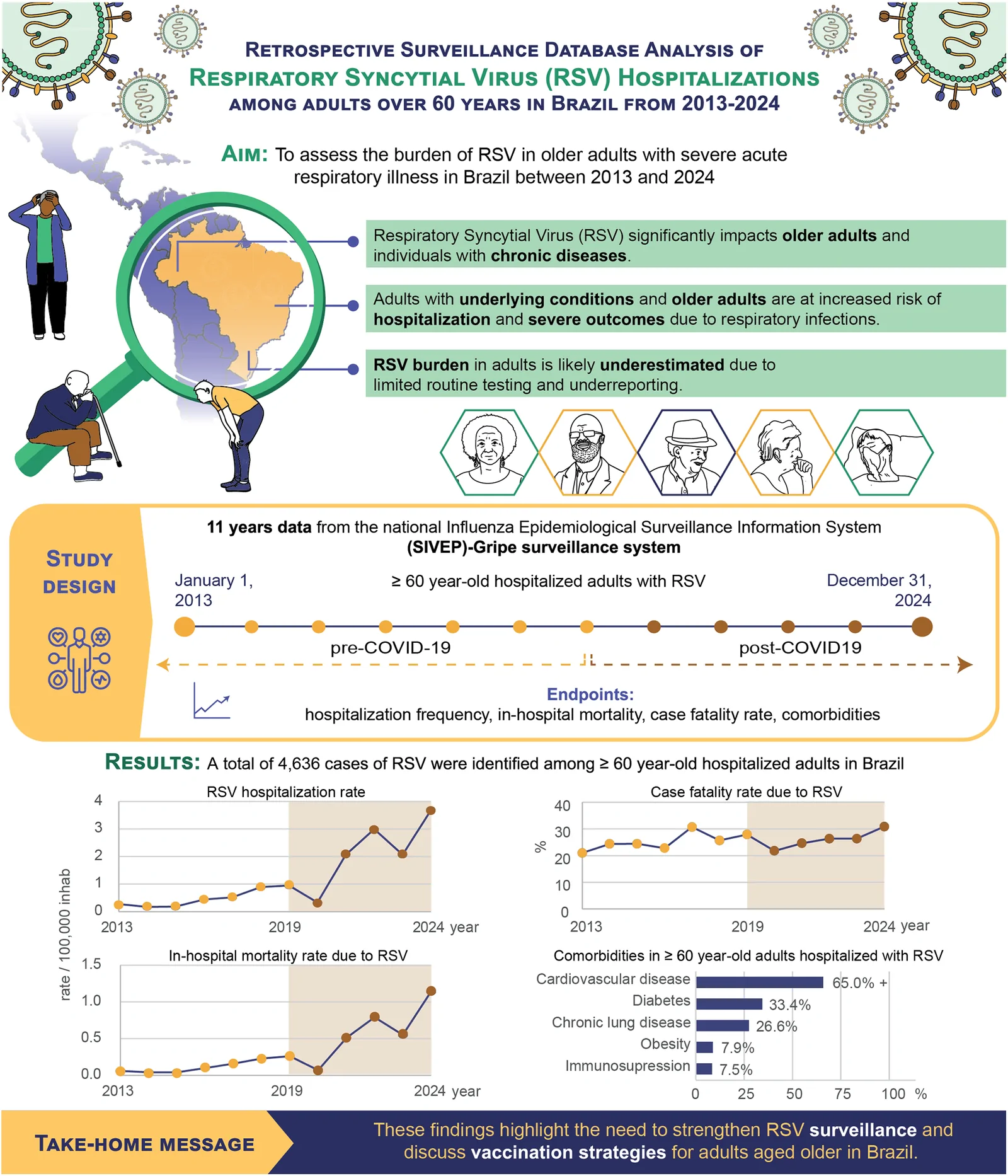
Graphical abstract.

Data on the RSV burden and associated outcomes are limited in adults aged ≥60 years in Brazil. The primary objective of this study was to determine the burden of RSV among SARI hospitalizations in adults aged ≥60 years in Brazil, using SIVEP-Gripe data. As secondary objective, we focused on describing healthcare resource utilization (HCRU), temporal trends and seasonality, and the presence of comorbidities among patients aged ≥60 years hospitalized with RSV-related SARIs. A graphical abstract highlighting the key outcomes of this study is included as Fig 1.

## Methods

### Study design and data source

This retrospective secondary database study used real-world data to evaluate the burden, temporal trends, seasonality, and HCRU of RSV-related SARI hospitalizations in Brazil. Data were extracted from the SIVEP-Gripe surveillance system on the 13 August 2024 and on 5 May 2025. The SIVEP-Gripe database provides freely available anonymized data accessible via OpenDataSUS portal [23]. The study included all hospitalized SARI cases reported between 1 January 2013 and 31 December 2024.

Analyses were stratified by year and by pre-COVID-19 (2013–2019), and post-COVID-19 (2020–2024) periods, to identify changes in seasonality and trends of RSV-related SARI hospitalizations.

### Study population and case definitions

According to Brazil’s national surveillance system, a SARI case is defined as a hospitalized patient experiencing an acute respiratory infection. This manifests with fever (self-reported or measured), cough or sore throat, dyspnea, oxygen saturation <95% or other breathing difficulties, and require hospitalization or death in the absence of an alternative diagnosis [24].

### Inclusion and exclusion criteria

Patients aged ≥60 years hospitalized with SARI and laboratory-confirmed RSV (diagnosed by RT-PCR or antigen testing), reported between 1 January 2013 and 31 December 2024 were considered eligible cases for inclusion in our analysis. If the same case presented more than one positive test, it was only counted as one case. SARI cases of viral etiology recorded in the SIVEP database are identified exclusively by molecular methods (RT-PCR) or antigen testing. We excluded cases for whom the diagnostic method was ambiguous.

### Study variables and outcomes

Primary outcomes included RSV-related SARI-hospitalization frequency and rate, in-hospital mortality rate, and CFR. Secondary outcomes considered were HCRU, temporal trends and seasonality, and the presence of comorbidities among RSV-related hospitalized SARI cases.

Outcomes were stratified by study period, age group, federative unit (Brazilian state), country region, healthcare system (private or public), and comorbidities. The comorbidities considered included cardiovascular diseases (heart failure, coronary artery disease, arterial hypertension, and other chronic heart diseases), chronic respiratory diseases (chronic obstructive pulmonary disease [COPD], asthma, interstitial lung disease, and other chronic pulmonary conditions), diabetes (type 1 and type 2), obesity (in Brazil, obesity is defined as a body mass index ≥30 kg/m^2^), and neurological diseases (stroke, Alzheimer’s disease, Parkinson’s disease, degenerative neurological diseases, and other chronic neurological conditions).

### Statistical analysis

Epidemiological profiles, time series, seasonality, and HCRU of RSV-related SARI hospitalizations were described. Continuous variables were summarized using measures of central tendency and dispersion. Categorical variables were presented as frequencies and proportions. Hospitalization rates were calculated per 100,000 population per year using projections from the Brazilian Institute of Geography and Statistics 2010 census. The in-hospital mortality is the number of deaths by the total population (per 100,000 population). The CFRs is the number of deaths divided by the number of hospitalized cases, multiplied by 100. These data were stratified by study period, region, and age group. Temporal projections were conducted using seasonal autoregressive integrated moving average models to account for the inherent seasonality and autocorrelation in RSV hospitalization data. Optimal model parameters were selected using the Akaike Information Criterion, ensuring a balance between goodness-of-fit and model complexity. Forecasts were generated for the years 2025 and 2026, based on historical patterns observed between 2013 and 2024, and stratified by age group and region to capture epidemiological variability [25].

All analyses were performed using Python v3.11.11. To compare severe RSV infection cases based on the presence and number of comorbidities and their association with the ICU admission rate, a logistic regression analysis was performed. Results are presented as odds ratios (ORs) with corresponding p-values. For comparisons involving annualized CFRs and length of hospital stay (in days), Dunn’s test with Bonferroni correction was applied to adjust for multiple comparisons and reduce the risk of a type I error, given the non-binary nature of these outcomes

### Ethical conduct of research

This study was a retrospective analysis of data from a publicly available database. Data are anonymized and de-identified. This study complies with all applicable laws regarding participant privacy. No direct subject contact or primary collection of individual human subject data occurred. Study results were in tabular form and aggregate analyses that prevent patient identification; therefore, informed consent, ethics committee, or Institutional Review Board (IRB) approval were not required.

## Results

### Epidemiological profile of RSV in hospitalized SARI cases

#### Demographic characteristics.

Between 2013 and 2024, there were 118,076 new SARI hospitalizations attributed to the RSV virus across all age groups reported via SIVEP-Gripe. Of these, 90.7% (107,146) of cases were in children aged 0–4 years. In adults, 2.2% (2,644) and 3.9% (4,636) of cases were in the 20–59 and ≥60 years age groups, respectively. For RSV-related SARI hospitalizations in adults aged ≥60 years, case frequency increased with age: 1,355 (29.2%) were aged 60–69 years, 1,522 (32.8%) were aged 70–79 years, and 1,759 (37.9%) aged ≥80 years. Women comprised the majority of cases (59.0%) among adults aged ≥60 years (Table 1). At the state level, Paraná reported the highest percentage of cases (23.2%), followed by São Paulo (15.6%), and Rio Grande do Sul (15.0%). Together, these states accounted for more than half of all hospitalizations in adults aged ≥60 years (Table 1). Data stratified by year are provided in S1 Table in S1 File.

**Table 1:** Demographic characteristics of patients aged ≥60 years hospitalized with RSV-related severe acute respiratory infection (SARI).

Demographic characteristic	Age group			
	**60-69 years**	**70-79 years**	**≥ 80 years**	**≥ 60 years**
**Gender** – n (%)				
n	1,355	1,522	1,759	4,636
Female	733 (54.1%)	860 (56.5%)	1,144 (65.0%)	2,737 (59.0%)
Male	622 (45.9%)	662 (43.5%)	615 (35.0%)	1,899 (41.0%)
				
**Ethnicity** – n (%)				
n	1,352	1,518	1,753	4,623
White	714 (52.7%)	883 (58.0%)	1,028 (58.4%)	2,625 (56.6%)
Black	58 (4.3%)	50 (3.3%)	45 (2.6%)	153 (3.3%)
Brown	397 (29.3%)	394 (25.9%)	415 (23.6%)	1,206 (26.0%)
Indigenous	3 (0.2%)	4 (0.3%)	6 (0.3%)	13 (0.3%)
Asian	12 (0.9%)	13 (0.9%)	21 (1.2%)	46 (1.0%)
				
**Region of residence** – n (%)				
n	1,355	1,522	1,759	4,636
North	78 (5.8%)	82 (5.4%)	88 (5.0%)	248 (5.3%)
Northeast	148 (10.9%)	144 (9.5%)	225 (12.8%)	517 (11.2%)
South	572 (42.2%)	698 (45.9%)	738 (42.0%)	2,008 (43.3%)
Southeast	420 (31.0%)	437 (28.7%)	559 (31.8%)	1,416 (30.5%)
Midwest	137 (10.1%)	161 (10.6%)	149 (8.5%)	447 (9.6%)
				
**State of residence** – n (%)				
n	1,355	1,522	1,759	4,636
Acre	20 (1.5%)	14 (0.9%)	13 (0.7%)	47 (1.0%)
Alagoas	6 (0.4%)	5 (0.3%)	6 (0.3%)	17 (0.4%)
Amazonas	25 (1.8%)	37 (2.4%)	41 (2.3%)	103 (2.2%)
Amapá	1 (0.1%)	0 (0.0%)	0 (0.0%)	1 (0.0%)
Bahia	62 (4.6%)	63 (4.1%)	84 (4.8%)	209 (4.5%)
Ceará	12 (0.9%)	18 (1.2%)	27 (1.5%)	57 (1.2%)
Distrito Federal	29 (2.1%)	22 (1.4%)	25 (1.4%)	76 (1.6%)
Espirito Santo	30 (2.2%)	22 (1.4%)	48 (2.7%)	100 (2.2%)
Goiás	31 (2.3%)	61 (4.0%)	48 (2.7%)	140 (3.0%)
Maranhão	5 (0.4%)	8 (0.5%)	8 (0.5%)	21 (0.5%)
Minas Gerais	114 (8.4%)	109 (7.2%)	151 (8.6%)	374 (8.1%)
Mato Grosso do Sul	74 (5.5%)	75 (4.9%)	73 (4.2%)	222 (4.8%)
Mato Grosso	3 (0.2%)	3 (0.2%)	3 (0.2%)	9 (0.2%)
Pará	14 (1.0%)	22 (1.4%)	18 (1.0%)	54 (1.2%)
Paraíba	20 (1.5%)	14 (0.9%)	32 (1.8%)	66 (1.4%)
Pernambuco	10 (0.7%)	17 (1.1%)	19 (1.1%)	46 (1.0%)
Piauí	19 (1.4%)	12 (0.8%)	20 (1.1%)	51 (1.1%)
Paraná	297 (21.9%)	377 (24.8%)	403 (22.9%)	1,077 (23.2%)
Rio de Janeiro	73 (5.4%)	71 (4.7%)	73 (4.2%)	217 (4.7%)
Rio Grande do Norte	6 (0.4%)	2 (0.1%)	7 (0.4%)	15 (0.3%)
Rondônia	10 (0.7%)	3 (0.2%)	6 (0.3%)	19 (0.4%)
Roraima	1 (0.1%)	0 (0.0%)	0 (0.0%)	1 (0.0%)
Rio Grande do Sul	207 (15.3%)	243 (16.0%)	244 (13.9%)	694 (15.0%)
Santa Catarina	68 (5.0%)	78 (5.1%)	91 (5.2%)	237 (5.1%)
Sergipe	8 (0.6%)	5 (0.3%)	22 (1.3%)	35 (0.8%)
São Paulo	203 (15.0%)	235 (15.4%)	287 (16.3%)	725 (15.6%)
Tocantins	7 (0.5%)	6 (0.4%)	10 (0.6%)	23 (0.5%)

RSV: respiratory syncytial virus

Among individuals aged ≥60 years, most reported hospitalizations occurred within the private healthcare system (S2a Table in S1 File).

### Hospitalization rates and frequencies

**Fig 2:**
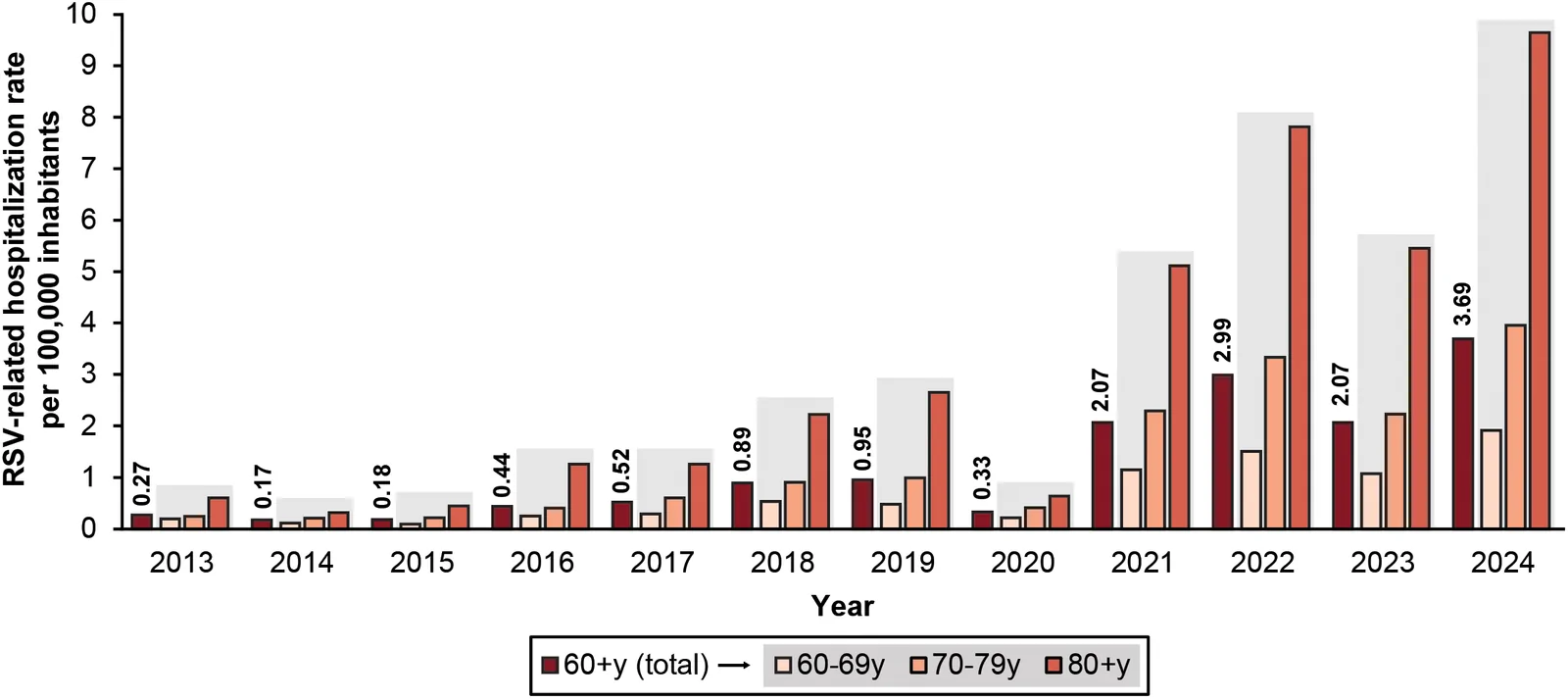
Yearly RSV-related hospitalization rates between 2013 and 2024 in adults aged ≥60 years. RSV: respiratory syncytial virus; y: years.

Among adults aged ≥60 years, RSV-related hospitalizations showed distinct patterns before and after the COVID-19 pandemic. In the pre-pandemic period (2013−2019), hospitalizations frequency increased by 142.1% between 2016 and 2019 (S1 Fig in S1 File). Corresponding hospitalization rates increased from 0.44 to 0.95 per 100,000 (S1 Fig in S1 File). A sharp decline was observed in 2020, with just 101 hospitalizations and a rate of 0.33 per 100,000, coinciding with the first year of the COVID-19 pandemic (Fig 2). In the post-pandemic years, a marked resurgence occurred. Hospitalizations number increased by 98.1% between 2021 and 2024, while hospitalization rates increased from 2.07 per 100,000 in 2021 to 3.69 per 100,000 in 2024 (Fig 2).

### In-hospital mortality rates

**Fig 3:**
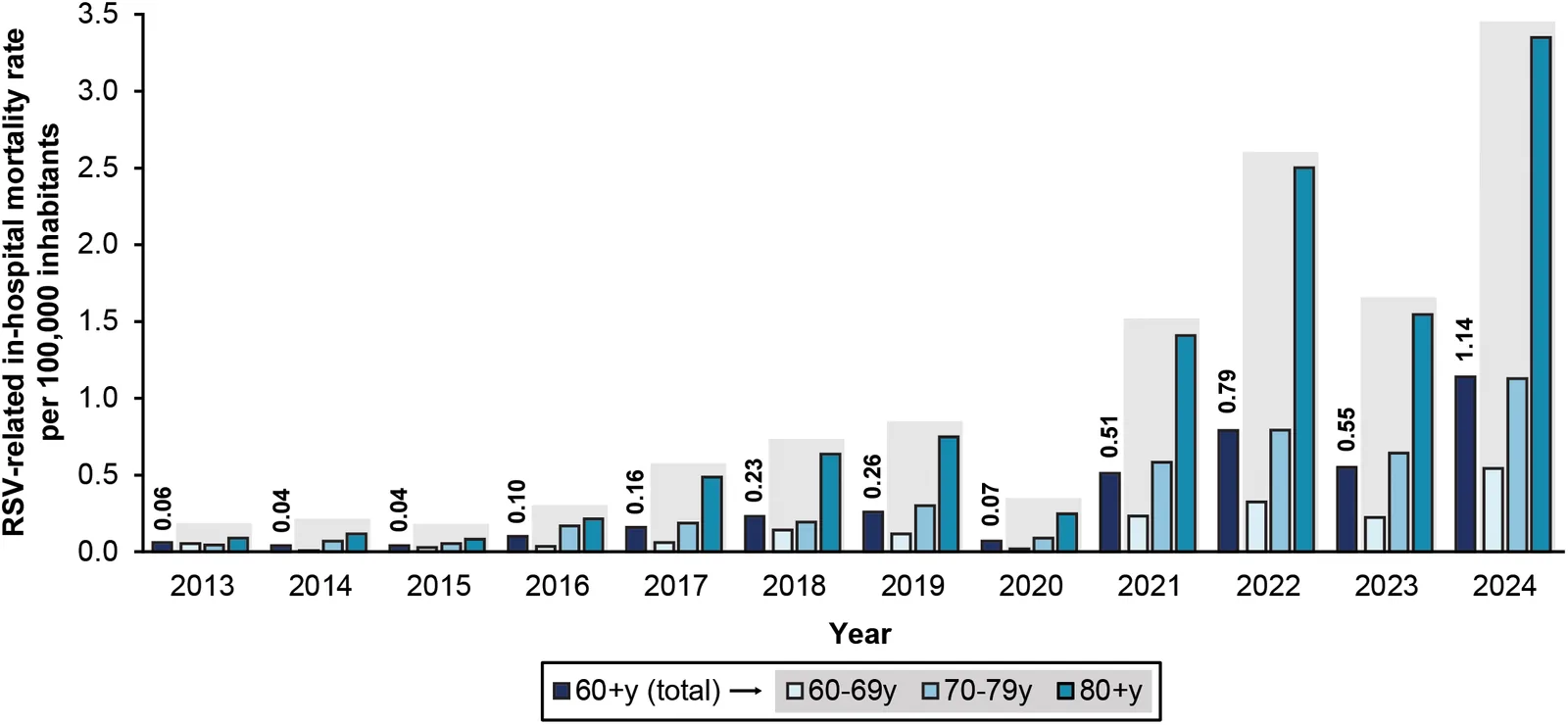
In-hospital mortality rate due to RSV among individuals aged ≥60 years. RSV: respiratory syncytial virus; y: years.

The in-hospital mortality rate due to RSV in individuals aged ≥60 years fluctuated between 2013 and 2024 but exhibited an increasing trend overall. Mortality rate increased from 0.06 (95% confidence interval [CI]: 0.03–0.09) in 2013 to 1.14 (95% CI: 1.03–1.25) in 2024 (Fig 3). The pre-pandemic rate was 0.13 (95% CI: 0.09–0.17), while the post-pandemic rate increased to 0.62 (95% CI: 0.52–0.71).

### Case fatality rate

**Fig 4:**
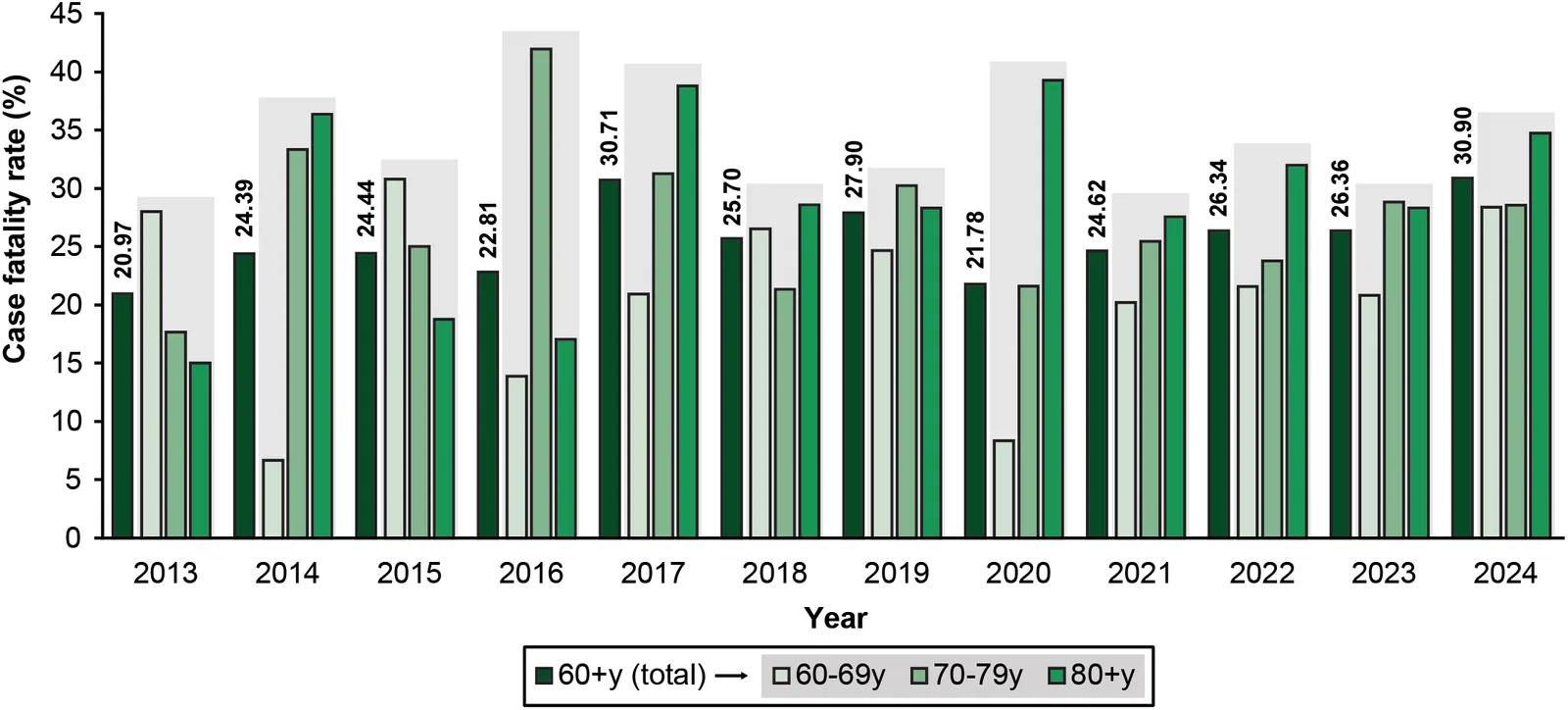
Case fatality rate due to RSV between 2013 and 2024 among adults aged ≥60 years. RSV: respiratory syncytial virus; y: years.

In adults aged ≥60 years, the RSV CFR varied over the years, with a gradual increase and notable peaks in 2024 (CFR: 30.9; 95% CI: 28.4–33.4), 2017 (CFR: 30.7; 95% CI: 23.1–38.4), 2019 (CFR: 27.9; 95% CI: 22.6–33.2) (Fig 4). The pre-pandemic CFR was 26.3 (95% CI: 18.8–33.8), and the post-pandemic CFR was 27.5 (95% CI: 24.3–30.7). For those aged 60–69 years, the CFR for RSV also varied, with peaks in 2015 (CFR: 30.8; 95% CI: 5.7–55.9), 2024 (CFR: 28.4; 95% CI: 23.7–33.0), and 2013 (CFR: 28.0; 95% CI: 10.4–45.6). The pre-pandemic CFR was 23.0 (95% CI: 7.0–39.0), and the post-pandemic CFR was 23.0 (95% CI: 14.3–31.8). In adults aged ≥80 years, the CFR for RSV was higher, with peaks in 2020 (CFR: 39.3; 95% CI: 21.2–57.4), 2017 (CFR: 38.8; 95% CI: 25.1–52.4), and 2014 (CFR: 36.4; 95% CI: 8.0–64.8). Comparing the periods, the pre-pandemic CFR was 27.4 (95% CI: 11.8–42.9), and the post-pandemic CFR was 31.7 (95% CI: 23.2–40.1).

For adults aged ≥60 years, the CFR for RSV ranged from 21.0% in 2013 to 30.9% in 2024. For adults aged ≥80 years, the CFR showed considerable variation, being higher than that observed in adults aged ≥60 years in most of the years analyzed, with the lowest CFR recorded of 15.0% in 2013, and notable peaks in 2017 (38.8%), 2020 (39.3%), and 2024 (34.7%).

### Healthcare resource utilization

For adults aged ≥60 years who were hospitalized due to RSV, the total number of hospitalizations was considerably higher in the post-pandemic period (3,709; 80.0%) compared with the pre-pandemic period (927; 20.0%). The mean length of hospital stay was slightly shorter in the post-pandemic period (12.5 days; SD: 16.0) than in the pre-pandemic period (14.2 days; SD: 19.0), with a median of 8.0 days in both periods. ICU admission was required in 31.9% of cases, with more admissions in the pre-pandemic period (385; 41.5%) than in the post-pandemic period (1,092; 29.4%). The mean length of stay in ICU was 12.1 days (SD 16.1), with a median of 7.0 days. This was slightly higher in the pre-pandemic period (13.9 days; SD: 18.8) than in the post-pandemic period (11.4 days; SD: 14.8). Ventilatory support was required in 69.0% of cases, with only slight variation between the pre-pandemic (638; 68.8%) and post-pandemic (2,563; 69.1%) periods (Table 2).

**Table 2:** Healthcare resource utilization by year among population individuals aged ≥60 years.

Year of admission	Total (N)	Number of hospitalizations, n (%)	Length of hospital stay, days; mean (SD)	Care unit use, n (%)	Length of intensive care unit stay, days; mean (SD)	Ventilatory support use, n (%)
**2013**	**1,480**	62 (1.3%)	11.7 (21.1)	18 (29.0%)	10.8 (7.3)	34 (54.8%)
**2014**	**1,372**	41 (0.9%)	11.1 (8.9)	13 (31.7%)	10.3 (12.5)	32 (78.0%)
**2015**	**1,697**	45 (1.0%)	11.1 (13.5)	15 (33.3%)	12.8 (17.0)	36 (80.0%)
**2016**	**2,640**	114 (2.5%)	14.8 (20.4)	57 (50.0%)	10.0 (11.4)	78 (68.4%)
**2017**	**3,167**	140 (3.0%)	13.4 (17.4)	53 (37.9%)	9.5 (9.3)	94 (67.1%)
**2018**	**5,525**	249 (5.4%)	16.2 (22.0)	106 (42.6%)	11.4 (12.0)	169 (67.9%)
**2019**	**6,227**	276 (6.0%)	14.0 (17.5)	123 (44.6%)	18.9 (25.8)	195 (70.7%)
**2020**	**1,237**	101 (2.2%)	9.6 (10.0)	36 (35.6%)	6.9 (6.1)	58 (57.4%)
**2021**	**14,214**	650 (14.0%)	12.6 (15.8)	167 (25.7%)	12.0 (15.0)	493 (75.8%)
**2022**	**18,996**	972 (21.0%)	11.9 (14.2)	282 (29.0%)	11.1 (13.1)	696 (71.6%)
**2023**	**28,246**	698 (15.1%)	13.9 (19.1)	221 (31.7%)	12.6 (15.6)	451 (64.6%)
**2024**	**33,253**	1,288 (27.8%)	12.5 (15.7)	386 (30.0%)	11.0 (15.7)	865 (67.2%)
**Overall**	**118,054**	4,636 (100.0%)	12.9 (16.7)	1,477 (31.9%)	12.1 (16.1)	3,201 (69.0%)
**Pre-COVID-19 pandemic**	**22,108**	927 (20.0%)	14.2 (19.0)	385 (41.5%)	13.9 (18.8)	638 (68.8%)
**Post-COVID-19 pandemic**	**62,693**	3,709 (80.0%)	12.5 (16.0)	1,092 (29.4%)	11.4 (14.8)	2,563 (69.1%)

SD: standard deviation

There was substantial missing data for the year 2018 on healthcare system type, (S1 Table in S1 File) as information from the National Registry of Health Establishments (CNES) was only incorporated into SARI notification forms from 2019 onward.

Overall, RSV-related hospitalizations showed differences between the public and private healthcare systems. The mean length of hospital stay was longer in public hospitals (10.1 days) than in private hospitals (8.6 days). Similarly, the mean length of ICU stays was shorter in the public system (10.6 days) than in the private system (8.2 days). A higher proportion of hospitalizations required ICU care in private hospitals (36.7%) than in public hospitals (24.9%). Ventilatory support was more frequently used in the public sector (69.7%) than in the private sector (65.5%) (S2b Table in S1 File).

In the post-pandemic period, hospitalizations increased markedly in both sectors (more than 93% of cases occurred after the onset of the pandemic). The mean length of hospital stay decreased compared with the pre-pandemic period in both private (10.4 to 8.4 days) and public hospitals (11.9 to 10.0 days). ICU admission rates also declined, from 43.9% to 36.2% in private hospitals and from 30.6% to 24.6% in public hospitals. Similarly, ICU stays were shorter post-pandemic (13.3 vs 7.6 days in private hospitals; 18.9 vs 9.7 days in public hospitals). Ventilatory support use also decreased, from 72.6% to 65.0% in private hospitals and from 77.7% to 69.2% in public hospitals.

Chest X-rays were performed in 40.7% of patients aged ≥60 years. The number of chest x-rays performed varied by age group, with 560 chest x-rays performed in individuals aged 60–69 years (41.3%), 642 in those aged 70–79 years (42,2%) and 685 (38.9%) in individuals aged ≥80 years (S3 Table in S1 File). Chest X-rays showed a spectrum of findings, ranging from consolidation (suggesting alveolar filling, as in pneumonia) and interstitial infiltrates (indicative of interstitial lung involvement) to mixed patterns combining both features, normal results, and other findings that did not fit these specific categories. For adults aged ≥60 years, 12.4% and 45.2% of the results showed consolidation and interstitial infiltrate, respectively.

### Temporal trends and seasonality

**Fig 5:**
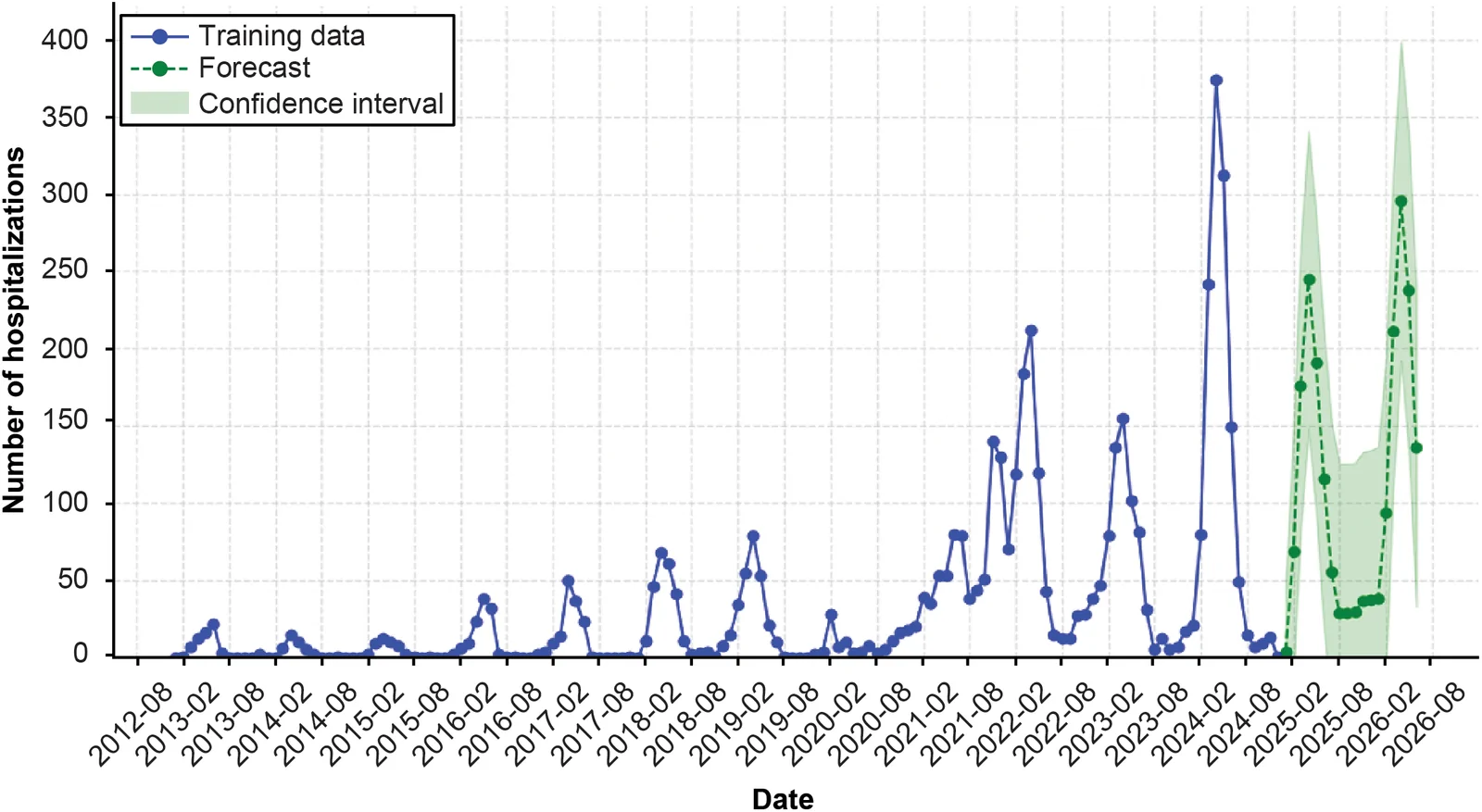
Time series of the number of monthly hospitalizations for RSV in adults aged ≥60 years between 2013 and 2024, with forecasts for 2025 and 2026. RSV: respiratory syncytial virus.

Time-series analyses were conducted to identify trends in RSV hospitalizations over the study period and make predictions about future trends based on these values. RSV hospitalizations among individuals aged ≥60 years exhibited a clear seasonal pattern. Peaks typically emerged in the first half of each year, with a notable decline in the second half (Fig 5). However, the COVID-19 pandemic greatly disrupted this pattern. In 2020, hospitalizations decreased dramatically, then gradually increased again in 2021. In 2022, hospitalizations surged, followed by a further decline in 2023. There was then another peak in 2024 (Fig 5). Due to these irregularities, the forecasts for 2025 and 2026 anticipate similar pattern of peaks in hospitalizations during the same period (Fig 5).

### Comorbidities

**Fig 6:**
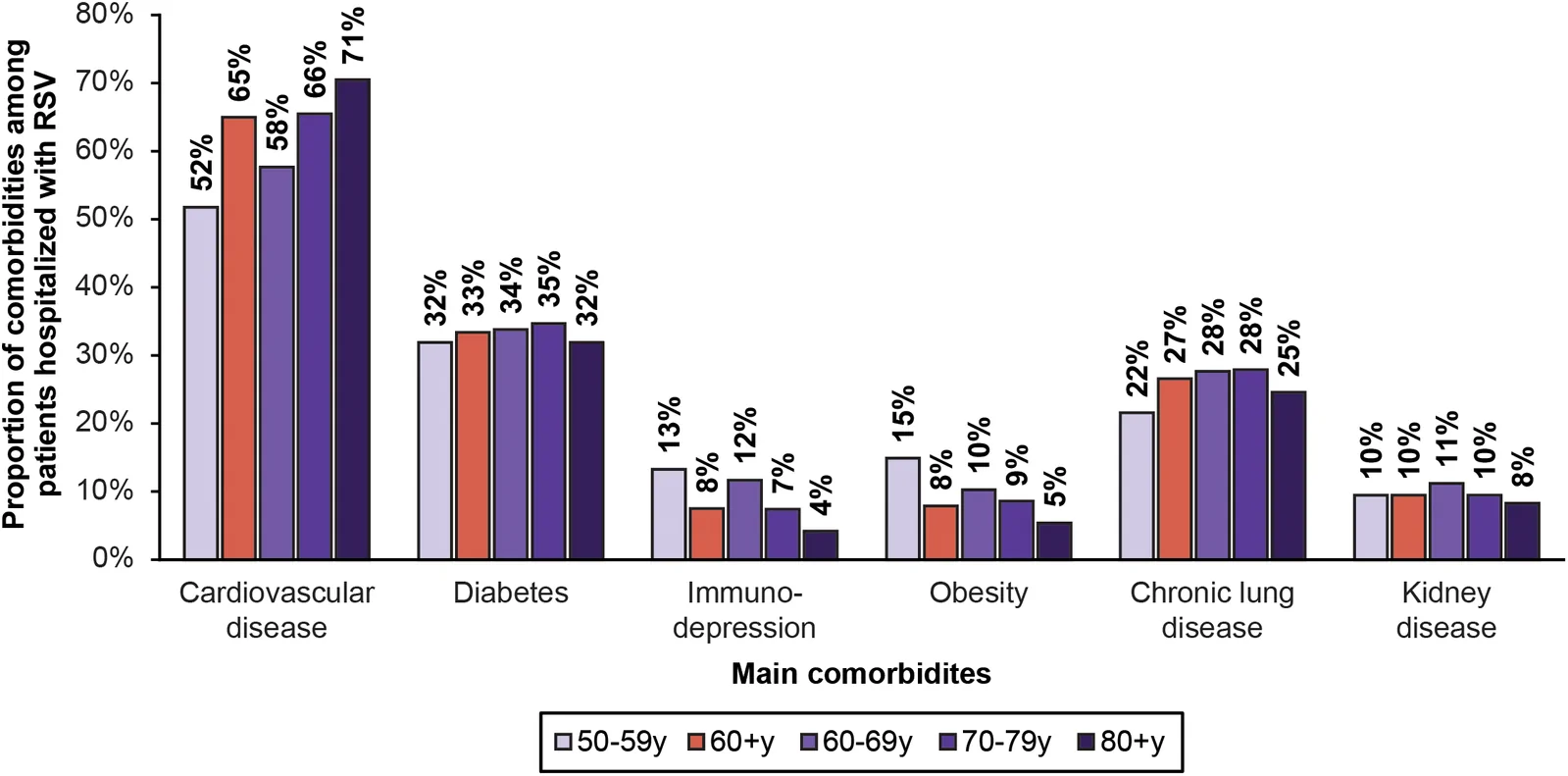
Main comorbidities in patients aged ≥50 years who were hospitalized with RSV. RSV: respiratory syncytial virus; y: years. Note: Other comorbidities in individuals aged ≥60 years were asthma (250; 7.5%), hematologic disease (74; 2.2%), chronic liver disease (60; 1.8%), metabolic disease (153; 4.6%), and neuropathy (394; 11.9%). Comorbidities data for those aged 50–59 years was available and hence, included in this figure.

Among individuals aged ≥60 years that were hospitalized with RSV, the majority of cases (71.5%) had at least one comorbidity. The most prevalent comorbidities included cardiovascular diseases (2,154; 65.0%), diabetes (1,108; 33.4%), and chronic lung diseases (882; 26.6%) (Fig 6).

Among adults aged ≥60 years with RSV-related hospitalizations, patients with at least one comorbidity required admission to the ICU more frequently than those without a comorbidity (34.6% vs 25.0%; odd ratio [OR]: 1.59, 95% CI: 1.38–1.83; p < 0.01) (Table 3). The need for ICU admission was significantly longer with the number of comorbidities, from 31.1% with one comorbidity to 43.9% with ≥4 comorbidities (OR: 2.35, 95% CI: 1.64–3.36; p < 0.01). The mean hospital stay was longer in patients with comorbidities (13.2 vs 12.2 days; p < 0.01), increasing to 17.9 days among those with ≥4 comorbidities. The CFR was also significantly higher in patients with comorbidities (28.4% vs 24.4%; p < 0.01) and increased with the number of comorbidities, from 26.5% with one comorbidity to 32.4% with ≥ 4 comorbidities. Dunn’s test confirmed that the differences in CFR were statistically significant for patients with ≥3 comorbidities, while differences in length of stay were significant for those with ≥4 comorbidities (Table 3).

**Table 3:** Presence of comorbidities among all adults aged ≥60 years who were hospitalized with RSV.

Variable	No comorbidities	At least one comorbidity	Number of comorbidities			
			One	Two	Three	Four
Intensive care unit (ICU) use	24.98%	34.60%	31.12%	36.33%	38.60%	43.88%
Length of stay in hospital (days)	12.18	13.16	12.24	13.60	13.64	17.87
Annualized CFR	24.38	28.42	26.45%	28.55%	33.00%	32.37%

RSV: respiratory syncytial virus

## Discussion

In this study, we assessed RSV-associated SARI hospitalizations and deaths in Brazil from 2013 to 2024, among adults aged ≥60 years. The results highlight a considerable disease burden within this age group, reveal notable regional disparities, and underscore ongoing gaps in surveillance systems that hinder precise estimates of the burden. A total of 4,636 RSV hospitalizations occurred among individuals aged ≥60 years in Brazil (SIVEP-Gripe data). Hospitalization rates increased steadily with advancing age, and women represented the most cases. Hospitalizations were clustered in the South and Southeast, with Paraná, São Paulo, and Rio Grande do Sul accounting for more than half of the cases. This pattern likely reflects regional disparities in healthcare access, diagnostic capacity, and surveillance completeness, as reported in Brazilian and Latin American studies [9,13].

The mean hospital stay was 12.9 days (median: 8.0), consistent with reports from Latin America and other regions where older adults experience prolonged admissions due to comorbidities [26]. Admission to ICU was necessary in 31.9% of cases, similar than that reported in a single-center adult cohort in Brazil (34.5%) [27]. RSV-related CFRs were substantially higher among older adults than in younger age groups and showed marked year-to-year variability, with peaks exceeding 30% in several seasons, particularly in those aged ≥80 years. In contrast, CFRs among children aged 0–4 years showed minimal variation over time, ranging from 1.11% in 2021 to 2.68% in 2013 (S2 Fig in S1 File). This age-specific pattern aligns with global evidence indicating that although infants experience the highest RSV mortality worldwide, older adults also bear a considerable and often underrecognized mortality burden [28]. Our findings confirm a high level of HCRU in this population [29,30].

During the COVID-19 pandemic, RSV case numbers decreased, with a sharp decrease in 2020, then numbers rebounded in subsequent years, with altered seasonal timing, an effect also reported elsewhere [31]. In our cohort, RSV hospitalizations among adults aged ≥60 years increased in the post-pandemic period but were characterized by shorter hospital stays and fewer ICU admissions, suggesting a possible attenuation in disease severity. Similar trends have been described in the US, with a study reporting shifts in the seasonality, age distribution, and clinical presentation of RSV following the relaxation of COVID-19 control measures [32]. However, a large cohort study in the US found that RSV hospitalized adults was as severe as COVID-19 or influenza in unvaccinated individuals and more severe than these infections in vaccinated patients for critical outcomes such as invasive mechanical ventilation or death [33]. These findings highlight the heterogeneity of RSV disease patterns and severity among older adults in the post-pandemic period. Most admissions occurred in patients with pre-existing conditions, and comorbidities substantially increased CFR and HCRU. We observed an increase in the average number of comorbidities with advancing age. Moreover, a higher number of comorbidities were correlated with higher CFR and greater HCRU. Cardiovascular disease was reported to be the most frequent comorbidity. Similar trends have been documented in high-income settings, where one-third of older adults with RSV require hospitalization and one-quarter require ICU admission [4,34]. A Brazilian study conducted at a hospital in São Paulo involving adults, including older patients, reported that 79.59% of RSV hospitalizations occurred in individuals with comorbidities [34]. Of these, 46% required ventilatory support and 9.8% ICU admission, while the mortality rate was 32.61% [34]. Our mortality findings align with other reports of Brazilian cohorts in which high mortality rates were seen in older adults with comorbidities [13,19].

Despite the reporting of RSV being mandatory in Brazil, substantial underreporting persists, particularly in the North and Northeast, where healthcare infrastructure is limited [13,35]. Diagnostic variability and changes in case definitions further affect data consistency. For example, in 2016, fever was removed as a required symptom for a diagnosis of RSV; this expanded case detection but reduced comparability over time [36,37].

RSV represents a major, underestimated cause of hospitalization and death among Brazilian adults aged ≥60 years [9,13]. In recent years, major advances in pharmacological prevention have transformed the landscape of RSV control, particularly for populations at increased risk, such as older adults and individuals with comorbidities [38]. Three vaccines targeting the prefusion F protein (PreF) have been approved by the US Food and Drug Administration and the European Medicines Agency and are now available worldwide. They are the adjuvanted RSVPreF3 vaccine, the bivalent RSVPreF vaccine, and an mRNA-based RSV vaccine, all indicated for older adults, with RSVPreF also indicated for maternal immunization to protect infants [39–44]. In parallel, the monoclonal antibody nirsevimab has emerged as a preventive option for infants, with a single dose during the RSV season providing long-lasting protection; it is recommended by the World Health Organization for broad infant immunization programs [45]. In Brazil, two vaccines and one monoclonal antibody are authorized for use. The RSVPreF3 vaccine was approved by ANVISA (the Brazilian Health Regulatory Agency) in December 2023 for adults aged ≥60 years and its indication was expanded in August 2025 to include adults aged ≥50 years who are at increased risk of RSV infection [39,46]. The bivalent RSVPreF vaccine is licensed in Brazil for adults aged 18–59 years at increased risk of RSV infection andadults aged ≥60 years, [47]. Additionally, since December 2025, the RSVPreF vaccine is included in the Brazilian National Immunization Program for maternal immunization, as a strategy to reduce pediatric cases [48]. The Brazilian Society of Immunizations recommends both vaccines for adults aged ≥50 years who have comorbidities and for all adults aged ≥70 years as part of the routine vaccination schedule [49]. Regarding passive immunization, nirsevimab was incorporated into Brazil’s National Immunization Program in February 2025 and will be made available to all premature newborns and children up to two years of age with comorbidities from 2026 [50].

Our study had some limitations, and the results should be interpreted with care. The primary limitation was that although the notification and testing of hospitalized SARI cases are mandatory, their practical implementation remains inconsistent, with considerable variations between regions of the country and age groups [9,13]. States with less developed healthcare infrastructure face more pronounced challenges, resulting in high rates of underreporting, particularly in the North and Northeast regions [13]. Secondly, positivity rate analyses were limited by the underreporting of negative test results and variable testing practices. It is also important to consider the differences in the sensitivity of diagnostic tests used for RSV, which may impact the accuracy of the findings [13]. Additionally, many non-mandatory fields in the SIVEP-Gripe database are missing a large amount of data, limiting the scope for more detailed analyses.

Accurate estimates of disease burden remain essential for guiding immunization strategies [51,52]. Strengthening surveillance will be critical in Brazil as it considers introducing the RSV vaccine into its National Immunization Program [53]. Our findings have the potential to increase the understanding of the impact of RSV on older adults in Brazil and underscore the importance of implementing targeted prevention strategies, including vaccination, to protect this vulnerable population.

## Supporting information

S1 File The file named “Supplementary materials.docx” includes the supplementary items listed below. Supplementary Table S1. Proportion of RSV positive results between 2013 and 2018 among hospitalized SARI patients in Brazil. Supplementary Table S2. (a) Demographic characteristics of RSV hospitalized cases by age group and healthcare sector; (b) Characteristics of RSV hospitalized cases by year and period of admission and by healthcare sector (all ages). Supplementary Table S3. Results of chest X-rays performed on patients with RSV by age group. Supplementary Fig S1. Annual frequency of RSV-related hospitalizations in adults aged ≥60 years, 2013–2024. Supplementary Fig S2. Comparison of CFR due to RSV between infants and adults aged ≥60 years.
(DOCX)
