# Supplementary material for: Burden of respiratory syncytial virus (RSV) hospitalizations among adults aged 60 years or more in Brazil: A retrospective surveillance database analysis (2013–2024)
Source: PLoS One. 2026 Sep 18;21(9):e0357303. doi: 10.1371/journal.pone.0357303 (PMC13588511; doi:10.1371/journal.pone.0357303)
Supplement: Supplementary file 1 [file pone.0357303.s001.docx]

# Supplementary materials

**Supplementary Table S1. Proportion of RSV positive results between 2013 and 2018 among hospitalized SARI patients in Brazil.**

|  | **Year of admission*** | | | | | |
| --- | --- | --- | --- | --- | --- | --- |
|  | **2013** | **2014** | **2015** | **2016** | **2017** | **2018** |
|  | **(N=36,563)** | **(N=18,995)** | **(N=14,552)** | **(N=54,379)** | **(N=29,557)** | **(N=47,726)** |
| **RSV positive results** - n (%) |  |  |  |  |  |  |
| Overall | 1,480 (4.05%) | 1,372 (7.22%) | 1,697 (11.66%) | 2,640 (4.85%) | 3,167 (10.71%) | 5,518 (11.56%) |
| **Region of residence:** |  |  |  |  |  |  |
| North | 101 (0.28%) | 38 (0.2%) | 18 (0.12%) | 87 (0.16%) | 232 (0.78%) | 358 (0.75%) |
| Northeast | 66 (0.18%) | 241 (1.27%) | 216 (1.48%) | 240 (0.44%) | 304 (1.03%) | 746 (1.56%) |
| South | 1,070 (2.93%) | 776 (4.09%) | 925 (6.36%) | 1,503 (2.76%) | 1,408 (4.76%) | 2,111 (4.42%) |
| Southeast | 231 (0.63%) | 306 (1.61%) | 505 (3.47%) | 658 (1.21%) | 880 (2.98%) | 1,418 (2.97%) |
| Midwest | 12 (0.03%) | 11 (0.06%) | 33 (0.23%) | 152 (0.28%) | 343 (1.16%) | 892 (1.87%) |

N=number of tests performed; n=number of RSV positive test results. *Data from 2019 onwards were not available

**Supplementary Table S2. (a) Demographic characteristics of RSV hospitalized cases by age group and healthcare sector ; (b) Characteristics of RSV hospitalized cases by year and period of admission and by healthcare sector (all ages).**

a

|  | **0 to 4 years** | **5 to 9 years** | **10 to 19 years** | **20 to 29 years** | **30 to 39 years** | **40 to 49 years** | **50 to 59 years** | **≥60 years**  **(Total)** | **60 to 69 years** | **70 to 79 years** | **≥80 years** |
| --- | --- | --- | --- | --- | --- | --- | --- | --- | --- | --- | --- |
| **RSV (N)** | **107,146** | **2,751** | **899** | **515** | **587** | **622** | **920** | **4,636** | **1,355** | **1,522** | **1,759** |
| **Healthcare sector** - n (%) |  |  |  |  |  |  |  |  |  |  |  |
| Private | 40,470 (37.8%) | 1,070 (38.9%) | 326 (36.3%) | 153 (29.7%) | 215 (36.6%) | 234 (37.6%) | 366 (39.8%) | 2,094 (45.2%) | 560 (41.3%) | 684 (44.9%) | 850 (48.3%) |
| Public | 52,376 (48.9%) | 1,428 (51.9%) | 446 (49.6%) | 229 (44.5%) | 235 (40.0%) | 242 (38.9%) | 365 (39.7%) | 1,868 (40.3%) | 577 (42.6%) | 625 (41.1%) | 666 (37.9%) |
| Missing | 14,300 (13.3%) | 253 (9.2%) | 127 (14.1%) | 133 (25.8%) | 137 (23.3%) | 146 (23.5%) | 189 (20.5%) | 674 (14.5%) | 218 (16.1%) | 213 (14.0%) | 243 (13.8%) |

N=overall number of RSV hospitalizations; n=number of RSV hospitalizations with valid healthcare sector data.

b

|  | **Overall** |  | | | | | |  | **Period of admission** | |
| --- | --- | --- | --- | --- | --- | --- | --- | --- | --- | --- |
|  |  | **2018** | **2019** | **2020** | **2021** | **2022** | **2023** | **2024** | **Pre-COVID-19 pandemic** | **Post-COVID-19 pandemic** |
|  | **(N=107,720)** | **(N=5,525)** | **(N=6,227)** | **(N=1,237)** | **(N=14,214)** | **(N=18,996)** | **(N=28,268)** | **(N=33,253)** | **(N=11,752)** | **(N=95,968)** |
| *n valid* | 42,237 | 3 | 2,807 | 461 | 5,451 | 7,954 | 12,133 | 13,428 | **-** | **-** |
| *missing CNES** | 65,483 | 5,522 | 3,420 | 776 | 8,763 | 11,042 | 16,135 | 19,825 | **-** | **-** |
| **Healthcare sector: private** |  |  |  |  |  |  |  |  |  |  |
| **Number of hospitalizations** | 44,928 (100.0%) | 3 (0.0%) | 2,915 (6.5%) | 478 (1.1%) | 5,773 (12.8%) | 8,519 (19.0%) | 13,070 (29.1%) | 14,170 (31.5%) | 2,918 (6.5%) | 42,010 (93.5%) |
|  |  |  |  |  |  |  |  |  |  |  |
| **Length of hospital stay (days)** |  |  |  |  |  |  |  |  |  |  |
| *n valid* | 42,237 | 3 | 2,807 | 461 | 5,451 | 7,954 | 12,133 | 13,428 | 2,810 | 39,427 |
| Mean (SD) | 8.6 (12.1) | 16.7 (13.3) | 10.4 (13.1) | 9.2 (12.9) | 8.1 (12.3) | 8.1 (10.4) | 8.4 (12.6) | 8.8 (12.2) | 10.4 (13.1) | 8.4 (12.0) |
| Median (min-max) | 6.0 (0.0 - 178.0) | 10.0 (8.0 - 32.0) | 7.0 (0.0 - 171.0) | 6.0 (0.0 - 163.0) | 5.0 (0.0 - 159.0) | 5.0 (0.0 - 173.0) | 6.0 (0.0 - 178.0) | 6.0 (0.0 - 178.0) | 7.0 (0.0 - 171.0) | 5.0 (0.0 - 178.0) |
| IQR | 6.0 | 12.0 | 7.0 | 7.0 | 5.0 | 6.0 | 6.0 | 6.0 | 7.0 | 6.0 |
|  |  |  |  |  |  |  |  |  |  |  |
| **care unit ICU use** - n (%) | 16,475 (36.7%) | 2 (66.7%) | 1,279 (43.9%) | 192 (40.2%) | 2,131 (36.9%) | 3,147 (36.9%) | 4,512 (34.5%) | 5,212 (36.8%) | 1,281 (43.9%) | 15,194 (36.2%) |
|  |  |  |  |  |  |  |  |  |  |  |
| **Length of stay in the ICU (days)** |  |  |  |  |  |  |  |  |  |  |
| *n valid* | 12,047 | 2 | 1,236 | 136 | 1,521 | 2,233 | 3,243 | 3,676 | 1,238 | 10,809 |
| Mean (SD) | 8.2 (10.4) | 29.5 (33.2) | 13.3 (17.5) | 7.3 (8.1) | 7.6 (8.9) | 7.8 (10.0) | 7.6 (8.7) | 7.4 (8.7) | 13.3 (17.5) | 7.6 (9.0) |
| Median (min-max) | 5.0 (0.0 - 156.0) | 29.5 (6.0 - 53.0) | 8.0 (0.0 - 156.0) | 5.0 (0.0 - 65.0) | 5.0 (0.0 - 118.0) | 5.0 (0.0 - 139.0) | 5.0 (0.0 - 119.0) | 5.0 (0.0 - 148.0) | 8.0 (0.0 - 156.0) | 5.0 (0.0 - 148.0) |
| IQR | 6.0 | 23.5 | 11.0 | 5.0 | 6.0 | 6.0 | 6.0 | 6.0 | 11.0 | 6.0 |
|  |  |  |  |  |  |  |  |  |  |  |
| **Ventilatory support use** n (%) | 29,413 (65.5%) | 3 (100.0%) | 2,116 (72.6%) | 246 (51.5%) | 3,835 (66.4%) | 5,697 (66.9%) | 8,310 (63.6%) | 9,206 (65.0%) | 2,119 (72.6%) | 27,294 (65.0%) |
| **Healthcare sector: public** |  |  |  |  |  |  |  |  |  |  |
| **Number of hospitalizations** | 57,189 (100.0%) | 4 (0.0%) | 3,305 (5.8%) | 756 (1.3%) | 8,424 (14.7%) | 10,464 (18.3%) | 15,191 (26.6%) | 19,045 (33.3%) | 3,309 (5.8%) | 53,880 (94.2%) |
|  |  |  |  |  |  |  |  |  |  |  |
| **Length of hospital stay (days)** |  |  |  |  |  |  |  |  |  |  |
| *n valid* | 54,321 | 4 | 3,223 | 664 | 8,040 | 9,963 | 14,340 | 18,087 | 3,227 | 51,094 |
| Mean (SD) | 10.1 (13.7) | 21.8 (9.6) | 11.8 (13.5) | 12.6 (20.1) | 9.7 (16.2) | 9.8 (13.5) | 10.4 (13.6) | 9.9 (12.5) | 11.9 (13.5) | 10.0 (13.7) |
| Median (min-max) | 6.0 (0.0 - 179.0) | 23.0 (10.0 - 31.0) | 8.0 (0.0 - 142.0) | 7.0 (0.0 - 175.0) | 5.0 (0.0 - 172.0) | 6.0 (0.0 - 179.0) | 6.0 (0.0 - 176.0) | 6.0 (0.0 - 176.0) | 8.0 (0.0 - 142.0) | 6.0 (0.0 - 179.0) |
| IQR | 7.0 | 12.75 | 9.0 | 8.0 | 7.0 | 7.0 | 7.0 | 7.0 | 9.0 | 7.0 |
|  |  |  |  |  |  |  |  |  |  |  |
| **care unit ICU use** - n (%) | 14,252 (24.9%) | 2 (50.0%) | 1,011 (30.6%) | 194 (25.7%) | 1,844 (21.9%) | 2,462 (23.5%) | 3,782 (24.9%) | 4,957 (26.0%) | 1,013 (30.6%) | 13,239 (24.6%) |
|  |  |  |  |  |  |  |  |  |  |  |
| **Length of stay in the ICU (days)** |  |  |  |  |  |  |  |  |  |  |
| *n valid* | 9,460 | 2 | 980 | 97 | 1,169 | 1,729 | 2,510 | 2,973 | 982 | 8,478 |
| Mean (SD) | 10.6 (13.0) | 18.5 (0.7) | 18.9 (21.4) | 8.9 (9.6) | 9.8 (13.5) | 9.4 (10.5) | 9.9 (11.3) | 9.6 (10.8) | 18.9 (21.3) | 9.7 (11.3) |
| Median (min-max) | 7.0 (0.0 - 177.0) | 18.5 (18.0 - 19.0) | 12.0 (0.0 - 177.0) | 7.0 (0.0 - 74.0) | 6.0 (0.0 - 141.0) | 7.0 (0.0 - 131.0) | 7.0 (0.0 - 136.0) | 7.0 (0.0 - 164.0) | 12.0 (0.0 - 177.0) | 7.0 (0.0 - 164.0) |
| IQR | 8.0 | 0.5 | 18.0 | 7.0 | 9.0 | 7.0 | 8.0 | 8.0 | 18.0 | 8.0 |
|  |  |  |  |  |  |  |  |  |  |  |
| **Ventilatory support use** n (%) | 39,852 (69.7%) | 3 (75.0%) | 2,568 (77.7%) | 456 (60.3%) | 5,857 (69.5%) | 7,411 (70.8%) | 10,473 (68.9%) | 13,084 (68.7%) | 2,571 (77.7%) | 37,281 (69.2%) |

*National Registry of Health Establishments [Cadastro Nacional de Estabelecimentos de Saúde (CNES)]

N=overall number of RSV hospitalizations ; n=number of RSV hospitalizations with valid healthcare sector data.

ICU: intensive care unit; IQR: interquartile range; SD: standard deviation

**Supplementary Table S3. Results of chest X-rays performed on patients with RSV by age group.**

| **Chest X-ray** | **0 to 4 years** | **5 to 9 years** | **10 to 19 years** | **20 to 29 years** | **30 to 39 years** | **40 to 49 years** | **50 to 59 years** | **≥60 years** | **60 to 69 years** | **70 to 79 years** | **≥80 years** |
| --- | --- | --- | --- | --- | --- | --- | --- | --- | --- | --- | --- |
|  |  |  |  |  |  |  |  |  |  |  |  |
| **Number of test performed** | 64,545 (60.24%) | 1,504 (54.67%) | 478 (53.17%) | 219 (42.52%) | 261 (44.46%) | 283 (45.50%) | 417 (45.33%) | 1,887 (40.70%) | 560 (41.33%) | 642 (42.18%) | 685 (38.94%) |
|  |  |  |  |  |  |  |  |  |  |  |  |
| **Result** |  |  |  |  |  |  |  |  |  |  |  |
| Consolidation | 4,654 (7.21%) | 171 (11.37%) | 41 (8.58%) | 33 (15.07%) | 19 (7.28%) | 36 (12.72%) | 52 (12.47%) | 235 (12.45%) | 72 (12.86%) | 77 (11.99%) | 86 (12.55%) |
| Interstitial infiltrate | 28,603 (44.31%) | 563 (37.43%) | 208 (43.51%) | 92 (42.01%) | 127 (48.66%) | 122 (43.11%) | 197 (47.24%) | 853 (45.20%) | 251 (44.82%) | 287 (44.7%) | 315 (45.99%) |
| Mixed | 2.585 (4.00%) | 62 (4.12%) | 26 (5.44%) | 27 (12.33%) | 17 (6.51%) | 24 (8.48%) | 33 (7.91%) | 141 (7.47%) | 53 (9.46%) | 44 (6.85%) | 44 (6.42%) |
| Normal | 13,061 (20.24%) | 292 (19.41%) | 103 (21.55%) | 46 (21.00%) | 46 (17.62%) | 46 (16.25%) | 51 (12.23%) | 269 (14.26%) | 77 (13.75%) | 92 (14.33%) | 100 (14.6%) |
| Other | 15,642 (24.23%) | 416 (27.66%) | 100 (20.92%) | 21 (9.59%) | 52 (19.92%) | 55 (19.43%) | 84 (20.14%) | 389 (20.61%) | 107 (19.11%) | 142 (22.12%) | 140 (20.44%) |

**Supplementary Fig S1. Annual frequency of RSV-related hospitalizations in adults aged ≥60 years, 2013-2024.**

RSV-related hospitalizations

| Year | 60+ | 60-69 | 70-79 | 80+ |
| --- | --- | --- | --- | --- |
| 2013 | 62 | 25 | 17 | 20 |
| 2014 | 41 | 15 | 15 | 11 |
| 2015 | 45 | 13 | 16 | 16 |
| 2016 | 114 | 36 | 31 | 47 |
| 2017 | 140 | 43 | 48 | 49 |
| 2018 | 249 | 83 | 75 | 91 |
| 2019 | 276 | 77 | 86 | 113 |
| 2020 | 101 | 36 | 37 | 28 |
| 2021 | 650 | 198 | 216 | 236 |
| 2022 | 972 | 269 | 328 | 375 |
| 2023 | 698 | 197 | 229 | 272 |
| 2024 | 1288 | 363 | 424 | 501 |

**Supplementary Fig S2. Comparison of CFR due to RSV between infants and adults aged ≥60 years.**

Case fatality rate (%)

| Year | 0 to 4 years | 60+ years | 80 years+ |
| --- | --- | --- | --- |
| 2013 | 2.68199 | 20.9677 | 15 |
| 2014 | 2.12431 | 24.3902 | 36.3636 |
| 2015 | 1.81932 | 24.4444 | 18.75 |
| 2016 | 1.26582 | 22.807 | 17.0213 |
| 2017 | 2.47262 | 30.7143 | 38.7755 |
| 2018 | 2.2104 | 25.7028 | 28.5714 |
| 2019 | 2.45678 | 27.8986 | 28.3186 |
| 2020 | 2.31388 | 21.7822 | 39.2857 |
| 2021 | 1.11155 | 24.6154 | 27.5424 |
| 2022 | 1.14875 | 26.3374 | 32 |
| 2023 | 1.30779 | 26.361 | 28.3088 |
| 2024 | 1.11778 | 30.9006 | 34.7305 |

CFR: case fatality rate
